# A Critique of the Drug Discovery and Phase 3 Clinical Programs Targeting the Amyloid Hypothesis for Alzheimer Disease

**DOI:** 10.1002/ana.24188

**Published:** 2014-07-02

**Authors:** Eric Karran, John Hardy

**Affiliations:** 1 Alzheimer's Research UKCambridge; 2 Reta Lila Weston LaboratoriesLondon; 3 Department of Molecular Neuroscience, University College LondonLondon, United Kingdom

In 1906, Alois Alzheimer described the neuropathology of the disease that was to bear his name.^1^ Subsequently, our understanding of Alzheimer disease (AD) has grown significantly. The autosomal dominant mutations to the amyloid precursor protein (APP), presenilin (PS) 1, and PS2 genes that cause early onset AD have been very informative, leading to the articulation of the amyloid cascade hypothesis.^2^–^4^ This hypothesis has been the basis for several disease-modifying therapeutic approaches for AD. This has been partly because it provided a coherent framework for understanding AD pathogenesis but also because several pharmacological approaches that targeted the amyloid peptide (amyloidocentric) were sufficiently well-founded scientifically to enter clinical development. In the past 5 years, there have been 6 amyloidocentric programs that completed phase 3 clinical testing. None met their primary outcome measures (Table1), although 1, solanezumab, showed encouraging results in a prespecified secondary outcome measure. This disappointing track record has brought into question the amyloidocentric therapeutic approach. This review will consider these programs from the following perspectives:

1. What was the hypothesis being tested?
2. Did the preclinical data offer support for the hypothesis?
3. Did the clinical program establish that the drug mediated the desired effect, and how robust were the phase 2 data that were used to progress to a phase 3 trial?
4. What did the phase 3 trials demonstrate?

**Table 1:** Outcomes of Phase 3 Clinical Trials of Amyloidocentric Drugs

Drug Name and Proposed Mechanism of Action	Phase 2 Results	Phase 3 Results
Tramiprosate, Aβ aggregation inhibitor.	58 mild–moderate AD patients randomized to 4 groups: placebo, 50, 100, 150mg/kg tramiprosate b.i.d. for 3 months. Drug mediated a significant lowering of Aβ42 in CSF samples.^21^	1,052 mild–moderate AD patients randomized to 3 groups: placebo, 100, 150mg/kg b.i.d. for 78 weeks. No significant effects on primary outcome measures on ADAS-cog and CDR-SB.^25^
Tarenflurbil, γ-secretase modulator.	210 mild–moderate AD patients randomized to placebo, 400, 800mg b.i.d. tarenflurbil for 12 months. Some evidence of an improvement ADCS-ADL at the 800mg b.i.d. dose.^46^	1,684 mild AD patients randomized to placebo, 800mg b.i.d. tarenflurbil for 18 months. No significant effects on primary outcome measures on ADAS-cog and ADCS-ADL.^47^
Semagacestat, γ-secretase inhibitor.	51 mild–moderate AD patients randomized to placebo, 100, 140mg o.d. semagacestat following dose escalation for a total duration of 18 weeks. Significant reduction in plasma Aβ40 peptide.^77^	2,600 mild–moderate AD patients randomized to placebo, 100, 140mg semagacestat o.d. for 76 weeks in 2 trials (ClinicalTrials.gov identifiers NCT00594568, NTC00762411). Trials were halted after interim analysis showed increased incidence of skin cancer and worsening of cognition and activities of daily living.^78^
Bapineuzumab, humanized monoclonal antibody directed at amino acids 1–5 of Aβ peptide. Amyloid plaque clearance mediated by microglial activation.	234 mild–moderate AD patients, randomized to placebo, 0.15, 0.5, 1.0, or 2.0mg/kg bapineuzumab i.v. infusions every 13 weeks for 78 weeks. Some evidence of an improvement in cognitive and functional endpoints in study completers and *APOE4* noncarriers.^106^	4,500 mild–moderate AD patients randomized to placebo and 0.5mg/kg i.v. every 13 weeks for 18 months in *APOE4* carriers, and randomized to placebo, 0.5, 1.0mg/kg i.v. every 13 weeks for 18 months in *APOE4* noncarriers in 4 trials (ClinicalTrials.gov identifiers lNCT00575055, NCT00574132, NCT00676143, NCT00667810). Trials were halted after completion of 2 trials demonstrated a failure to meet primary outcome measures on ADAS-cog and activities of daily living.^109^
Solanezumab, humanized monoclonal antibody directed at amino acids 16–24 of Aβ peptide. Amyloid plaque clearance mediated via peripheral sink mechanism.	52 mild–moderate AD patients were randomized to placebo, 100mg every 4 weeks, 100mg weekly, 400mg every 4 weeks, 400mg weekly i.v. solanezumab for 12 weeks. There was a significant dose-dependent increase in Aβ42 peptide in CSF.^132^	2,000 mild–moderate AD patients randomized to placebo and 400mg solanezumab monthly i.v. for 18 months (ClinicalTrials.gov identifiers NCT00905372, NCT00904683). Trials failed to meet their primary outcome measures on ADAS-cog and ADCS-ADL. A secondary analysis of mild AD patients pooled from both trials showed a significant effect on cognition.^115^
Gammagard, intravenous immunoglobulin.	55 mild–moderate AD patients randomized to placebo, 0.2, 0.5, 0.8g/kg/4 weeks, or 0.1, 0.25, 0.4g/kg/2 weeks for 24 weeks. There was no increase in Aβ40 peptide in plasma at any dose.^129^	Trial data currently unpublished. 390 mild–moderate AD patients randomized to 0.2g/kg/2 weeks and 0.4g/kg/2 weeks vs placebo for 18 months (ClinicalTrials.gov Identifier NCT00818662). Gammagard failed to reach its coprimary outcomes of ADAS-cog and ADCS-ADL.

AD = Alzheimer disease; ADAS-cog = Alzheimer's Disease Assessment Scale–Cognitive Subscale; ADCS-ADL = Alzheimer's Disease Cooperative Study–Activities of Daily Living Inventory; b.i.d. = twice daily; CDR-SB = Clinical Dementia Rating–Sum of Boxes; CSF = cerebrospinal fluid; i.v. = intravenous; o.d. = once per day.

AD is responsible for approximately 70% of all dementias.^5^ Currently, a confirmed diagnosis of AD requires the presence of plaques (deposited amyloid β [Aβ] peptide) and tangles (intracellular, aggregated, hyperphosphorylated, tau protein) found via postmortem neuropathological examination of the brain. Although there are many abnormalities within an AD brain, neuronal death, particularly within the hippocampus, entorhinal cortex, and frontal cortical regions, contribute to cognitive impairment. The amount and regional distribution of plaques in AD brains does not correlate well with the extent of neuronal loss or with the clinical severity of dementia.^6^ There have been studies suggesting a better correlation with soluble Aβ in AD brain.^7^ However, deposited Aβ comprises approximately 95% of total Aβ (soluble plus deposited).^8^ The role of Aβ in AD has been long debated; does it trigger the disease process, is there some threshold amount that is required to sustain the disease, or does deposited Aβ drive the disease forward in a continuous fashion?^9^ The presence of tau pathology, in the form of insoluble paired helical filaments (PHFs), correlates much better both with the areas of the brain that suffer from neurodegeneration and also with the extent of cognitive impairment.^10^,^11^ However, the numbers of PHFs do not account for all the neuronal loss.^6^ Finally, brain volume remains the best pathological correlate of dementia in AD.^12^

## Drug Discovery and Development Programs

### Tramiprosate

#### What Was the Hypothesis Being Tested?

There is extensive literature demonstrating that proteoglycans bind to Aβ peptide and can accelerate the transition of soluble Aβ to a β-sheet structure that is required for the formation of plaques.^13^,^14^ Tramiprosate (3-amino-1-propanesulfonic acid) is a glycosaminoglycan mimetic that was discovered in a screen that measured the heparin-stimulated conversion of soluble Aβ40 from a random coil to the β-sheet structure that is characteristic of aggregated Aβ.^15^ Tramiprosate was tested for its ability to bind to soluble Aβ and thereby prevent its aggregation. Mechanistically, this would prevent the accumulation of aggregated Aβ and increase the levels of soluble Aβ in AD brain.

#### Did the Preclinical Data Offer Support for the Hypothesis?

The published data on tramiprosate are not as comprehensive as might be expected for a clinical candidate. A 20-fold molar excess of tramiprosate prevented the conversion of Aβ40 from random coil/alpha helix to β-sheet, as assessed by circular dichroism spectral analysis. No data were available for Aβ42.^16^ Experiments to determine the interaction between tramiprosate and Aβ were performed using electrospray mass spectrometry analysis, which provided evidence that tramiprosate was able to bind to both Aβ40 and Aβ42 with a 10-fold molar excess of drug required to give 50% binding, a finding that was replicated by others.^17^ However, there are no data on binding affinity or dose–response relationships, and the concentration of Aβ and tramiprosate used were very high for these experiments: 20μM of Aβ42 and 200μM of tramiprosate. This contrasts with a concentration of soluble Aβ42 in human cerebrospinal fluid (CSF) of ∼45pM.^18^ Furthermore, the relevance of this method to an aqueous phase system is not known. A more relevant approach was taken where Aβ42 was coated onto microtiter plates and test compounds together with fluorescently labeled Aβ42 were added to assess the potency of tramiprosate to prevent Aβ aggregation.^19^ Key elements of this assay were validated using fresh-frozen brain slices taken from AD brains. In this assay, inhibitory concentration of 50% (IC_50_) values of test compounds required to block aggregation of 0.22pM of Aβ were calculated. Tramiprosate was shown to be inactive at the highest concentration tested (718.6nM); that is, at a 3.2 × 10^6^ molar excess over Aβ. Using similar concentrations of Aβ and tramiprosate as had been used in the mass spectrometry analysis, but conducting the experiment in the aqueous phase, also failed to demonstrate any activity.^20^ At a 20-fold molar excess, tramiprosate was able to inhibit the cell death caused by 5μM Aβ42 applied to primary rat neurons.^16^ These data are difficult to interpret; there were no dose–response data, and the protective mechanism was not explored, so it is not possible to determine whether this effect was associated with inhibition of Aβ aggregation.

In 8-week-old TgCRND8 mice that carry the human APP K670N/M671L and V717F mutations, tramiprosate was administered subcutaneously (s.c.) daily at 30 or 100mg/kg for 8 weeks.^16^ The levels of compound in the brain at the end of this dosing regimen were not assayed. In a separate experiment, continuous infusion of ^14^C-tramiprosate for 10 days was used to estimate brain and plasma levels of drug in rats. At 2 doses, 1 and 10mg/kg/h, tramiprosate demonstrated brain drug levels of about 1μg/ml (70nM) and 10μg/ml (700nM), respectively. The drug half-life was between 2 and 4 hours in plasma and ≥16 hours in brain. However, the concentrations of total rather than free drug were assayed, and it is not known how these data might compare with the s.c. bolus administration that was used to determine efficacy.

The efficacy experiment demonstrated a significant effect on the percentage of the cortex occupied by plaques at 100mg/kg but not at 30mg/kg, and the drug had no effect on the number of thioflavin S–positive plaques at either dose. A more complete analysis would have required a wider range of doses and using one mouse brain hemisphere for histology and the other for quantitative biochemical analysis of Aβ species. Surprisingly, the levels of soluble plasma Aβ40 and Aβ42 were both reduced in a dose-related manner by tramiprosate. A reduction of circulating levels of Aβ is consistent with some type of facilitated clearance mechanism, although this was not explored further. A different cohort of TgCRND8 mice were bred that for unknown reasons showed a 4- to 5-fold increase in cerebral Aβ levels. In these mice, 9-week administration of 500mg/kg/day tramiprosate (a much larger dose) resulted in significant reductions in brain of both soluble and insoluble Aβ40 and Aβ42 peptides, data that are difficult to reconcile for an antiaggregation mechanism.

The preclinical data provided some support for an effect of tramiprosate on Aβ levels in brain, but the data were incomplete. An experimental design that incorporated a range of drug doses, mice analyzed at different times, histological and biochemical analysis performed simultaneously, and analysis of free and total drug levels would have provided a clearer picture of the therapeutic potential of the drug. Target engagement was not assayed; there was no detection of Aβ/tramiprosate complexes. The use of different doses in mice for histology and biochemistry, and the use of different mouse Aβ phenotypes for the 2 experiments, do not assist interpretation. These data would have confirmed or refuted the mechanistic hypothesis that at the administered doses tramiprosate binds to Aβ and prevents aggregation.

#### Preclinical to Clinical Translation

In the phase 2 program, tramiprosate was administered at 50, 100, and 150mg twice daily (b.i.d.) for 3 months to mild–moderate AD patients with a Mini-Mental State Examination (MMSE) scores between 13 and 25.^21^ It is not possible to determine how these doses were computed from the preclinical studies.

#### Did the Clinical Program Establish That the Drug Was Mediating the Desired Effect, and How Robust Were the Phase 2 Data That Were Used to Progress to a Phase 3 Trial?

Tramiprosate exposure did not increase in a dose-proportional manner between the 100 and 150mg b.i.d. dosing regimens. At 5 hours postdose, CSF samples were taken, and tramiprosate could be detected in ∼60% of patients at between 18 and 50nM. The concentration of total Aβ in CSF is approximately 1nM,^22^ and thus it is likely that tramiprosate achieved the 20-fold molar excess demonstrated to be required to bind to Aβ in some of the in vitro studies. However, it was not demonstrated whether tramiprosate/Aβ complexes were found in the CSF. Furthermore, some data suggest that Aβ concentrations in the extracellular space in the brain parenchyma might be as much as 100-fold greater than that found in CSF,^9^ which would mean that efficacious levels of tramiprosate may not have been achieved.

Nonetheless, there was a striking dose-dependent reduction in CSF Aβ42 levels of up to 70% after 3 months of treatment, with greater reductions seen in the mild AD population. If this reduction were seen in a therapeutic approach that was designed to inhibit Aβ production, it would have been an encouraging sign of efficacy and proof of mechanism. In AD, a reduction in CSF Aβ42 is interpreted as heralding an increase in Aβ42 deposition.^23^,^24^ Thus, an agent designed to prevent aggregation should elevate Aβ42 CSF levels to the normal range, unless the therapeutic agent acts both to prevent aggregation and to increase clearance or degradation. Furthermore, there was no effect on CSF Aβ40 levels, yet the preclinical in vitro data had shown no difference in the binding potential between Aβ40 and Aβ42. Tramiprosate had no effects on cognitive and clinical assessments, which is unsurprising given the short duration of the trial. The biomarker effects on CSF Aβ42 were considered sufficiently interesting to promote the Alphase phase 3 trial.

#### Tramiprosate Phase 3 Trial

Alphase was a double-blind, placebo-controlled multicenter study that enrolled 1,052 patients in North America and Canada.^25^ Tramiprosate was administered at 100mg b.i.d. and 150mg b.i.d. for 78 weeks. The primary endpoint measures were the Alzheimer's Disease Assessment Scale–Cognitive Subscale (ADAS-cog) and Clinical Dementia Rating–Sum of Boxes (CDR-SB). The study was powered to detect a 25% reduction in clinical deterioration. Hippocampal volume changes were assessed by magnetic resonance imaging (MRI) and used as a measure of disease modification. Unfortunately, this trial failed its primary and secondary endpoints. For unknown reasons, there was a significant variance introduced at different clinical trial sites that confounded the prespecified statistical analysis. Post hoc analysis showed some evidence of reduced hippocampal volume loss. Given that a surprising feature of the phase 2 data was a reduction in Aβ42 in the CSF, it is regrettable that these data are not available from the Alphase study. Tramiprosate is currently marketed as an over-the-counter supplement, Vivimind, for memory improvement.

### Tarenflurbil (R-Flurbiprofen)

#### What Was the Hypothesis Being Tested?

Epidemiological data suggest that the use of nonsteroidal anti-inflammatory drugs (NSAIDs) may offer some protection against the onset of AD,^26^ especially longer-term use,^27^,^28^ although this has not been seen by others.^29^–^31^ Interventional studies have been negative.^32^

However, anti-inflammatory agents were tested for their ability to affect Aβ production,^33^ and remarkably several commonly prescribed NSAIDs reduced Aβ42. Sulindac, indomethacin, and ibuprofen reduced the production of Aβ42, and this suppression was compensated for by an increase in the shorter Aβ metabolites, especially Aβ38. This work opened a new field of pharmacological intervention: the γ-secretase modulators. These agents are not inhibitors of γ-secretase, but shift the cleavage sites in favor of the production of shorter forms of Aβ. Most importantly, they do not affect the processing of an important substrate of γ-secretase, Notch.^34^ The effects on Aβ42 production were not mediated via inhibition of the NSAIDs' primary pharmacological target, the cyclooxygenase (COX) enzymes COX1 and COX2. It was shown that flurbiprofen racemate and the S- and R- enantiomers were equipotent,^35^ allowing use of R-flurbiprofen enantiomer (a less active COX inhibitor), thus reducing unwanted side effects, especially gastrointestinal toxicity. The hypothesis being tested was that R-flurbiprofen, subsequently named tarenflurbil, would provide a disease-modifying therapeutic agent for AD by reducing the production of Aβ42 in the brains of AD patients.

#### Did the Preclinical Data Offer Support for the Hypothesis?

The original published work on tarenflurbil^35^ did not establish full in vitro dose responses for inhibition of Aβ42 production. Other workers have used photoaffinity ligands attached to tarenflurbil and demonstrated that these were able to bind to an APP-derived substrate, but not to components of γ-secretase itself.^36^ However, subsequent studies have shown that tarenflurbil most likely binds allosterically to the γ-secretase complex^37^–^39^ that mediates a change in spectrum of Aβ metabolites in favor of shorter species^39^–^41^ with a median effective concentration (EC_50_) of Aβ42 inhibition of ∼250μM. Although the rationale of using tarenflurbil to reduce the potential side effect liability of COX inhibition has been widely accepted, it remains a more potent COX1 inhibitor (IC_50_ = 44μM) than an inhibitor of Aβ42 production.^42^ Thus, doses of tarenflurbil that suppressed Aβ42 production would always have COX1 suppression as a potential liability, or as an additional mechanism of efficacy, depending on the context.

**Figure 1:**
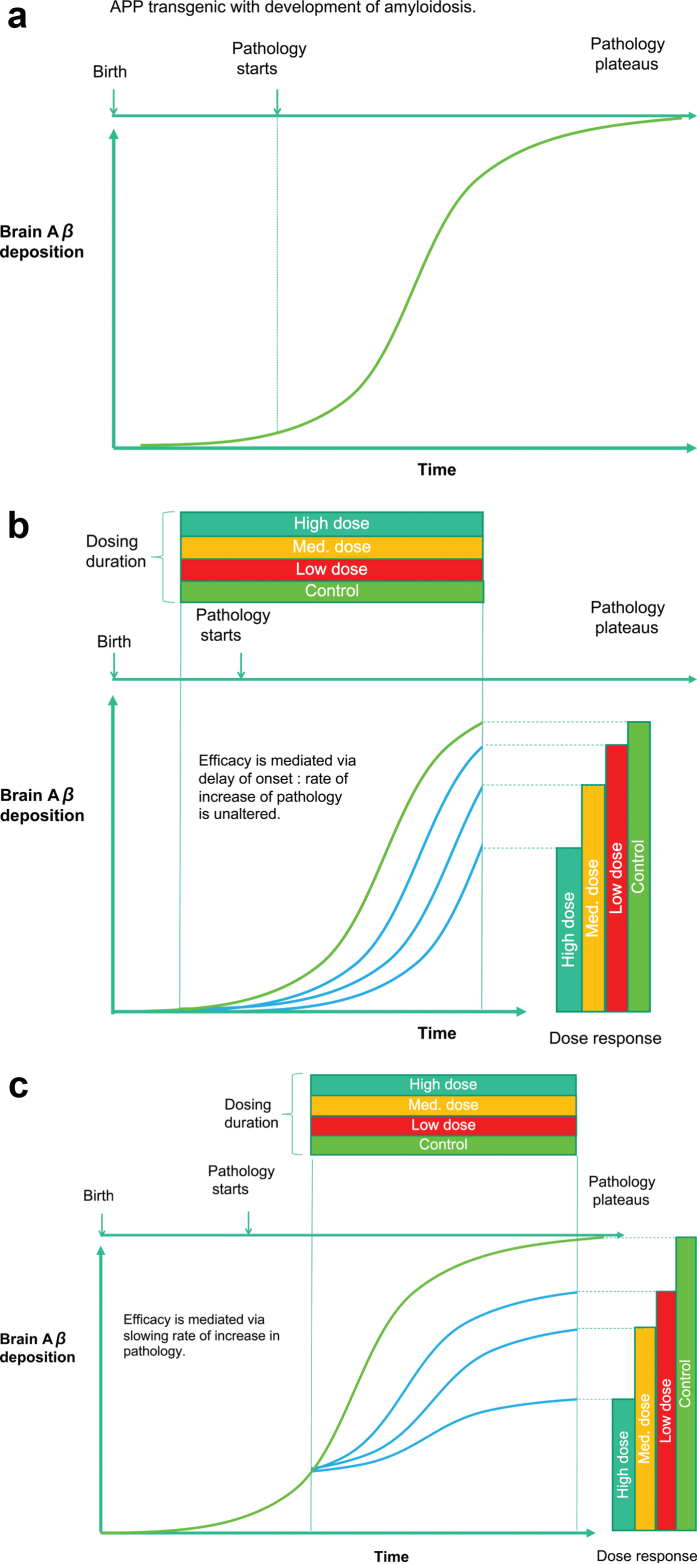
(A) The time course of the development of amyloid plaque in a typical APP transgenic mouse model. (B) Preventative paradigm. A potential amyloidocentric therapeutic agent is administered with dosing starting prior to the onset of amyloidosis. The therapeutic acts to delay the initial amyloid seeding events in a concentration-dependent manner but does not affect the rate of amyloid deposition. (C) Therapeutic paradigm. A potential amyloidocentric therapeutic agent is administered with dosing starting after the onset of amyloidosis. The therapeutic agent acts to slow the rate of amyloid deposition in a concentration-dependent manner. The dose responses of the therapeutic agent are very similar in B and C, but are potentially mediated via different mechanisms, and their construct validity in regard to the clinical situation has to be carefully considered.

The first publication of in vivo pharmacology was not comprehensive; 3 doses (10, 25, and 50mg/kg) of tarenflurbil were administered for 3 days to Tg2576 mice with levels of brain Aβ42 and Aβ40 measured.^35^ All 3 doses showed a reduction, but there was no dose response and the group sizes were low, ranging from 4 to 7 mice per group. The brain and plasma levels of tarenflurbil also did not increase in a dose-proportional manner. The measured brain levels of tarenflurbil were between 1.5 and 2.6μM, some 100-fold lower than the in vitro EC_50_ concentration. This discrepancy makes the suppression of Aβ42 levels difficult to interpret. A follow-up study in TG2576 mice looked at a longer-term dosing "preventative" paradigm (Fig 1), where 2 parallel groups were dosed at 10mg/kg tarenflurbil for 4 months between the ages of 8–9 and 11.5–12 months.^43^ Another group was dosed for 2 weeks from 17.5–18 to 18–19 months. The brains of the mice were analyzed using enzyme-linked immunosorbent assays (ELISAs) for Aβ40 and Aβ42 levels after formic acid and detergent extraction. Plaque burden was measured using immunohistochemistry. None of the treatment paradigms reduced Aβ42 or Aβ40. Surprisingly, plaque burden was reduced in the therapeutic paradigm but not the preventative paradigm. The discrepancy between a lack of effect on quantitative measurements of Aβ42 and Aβ40, and a significant lowering of plaque burden in the group that received just 2 weeks of tarenflurbil versus 4 months administration in the preventative group, makes these experiments difficult to interpret. Brain analysis of tarenflurbil and S-flurbiprofen showed evidence of significant enantiomeric biotransformation, and the total flurbiprofen concentration was 1μM, about 250-fold lower than the in vitro EC_50_. Other workers delivered tarenflurbil in food to 4- to 5-month-old Tg2576 mice at an average dose of 32mg/kg/day for 9 days.^41^ The study duration was cut short due to toxicity, which reduced the group size to N = 5. The tarenflurbil brain concentration was 1.3μM. There was a reduction in brain Aβ40, but not Aβ42, which increased compared to the control group. Given the very low N, and evidence of toxicity, interpretation of this study is challenging. Another study examined Aβ42 and Aβ40 levels in the cortex and hippocampus of 7- to 8-month-old Tg2576 mice following extraction with guanidinium HCl.^44^ The mice received 25, 50, and 100mg/kg flurbiprofen for 3 days. There were no effects on Aβ42 and Aβ40. A second experiment at lower doses of 10 and 25mg/kg showed a significant reduction in Aβ40 in the cortex but not in the hippocampus; Aβ42 was unaffected in both brain regions at both doses. Finally, another group administered 25mg/kg/day to 7- to 8-month-old Tg2576 mice for 7 days. There was no inhibition of brain Aβ42 or Aβ40 levels extracted using guanidinium HCl.^42^

In summary, the preclinical science identified a new pharmacological approach to the suppression of Aβ42 production. The in vitro data provided evidence for suppression of Aβ42 from cells with an EC_50_ of ∼250μM, although a clear modulator effect—a suppression of Aβ42 coupled to an increase in shorter forms of Aβ—was not always demonstrated. The in vitro EC_50_ concentration of tarenflurbil was never approached in the brain in the in vivo experiments due to the lack of brain penetration (about 1.5% of plasma levels^42^) and dose-limiting toxicity. A dose response of Aβ42 suppression, coupled to brain drug levels that were consistent with the EC_50_, was not demonstrated.

#### Preclinical to Clinical Translation

The clinical development of tarenflurbil appears to have been based on the few preclinical experiments that showed a reduction in brain Aβ42 levels. In a phase 1 study, 3 cohorts of 16 healthy aged subjects received either 400, 800, or 1600mg/day (n = 12, administered in 2 doses) or placebo (n = 4) for 21 days.^45^ The drug was well tolerated at all doses, and there was a dose-proportional increase in tarenflurbil concentrations in the CSF. At the highest dose, 800mg b.i.d., the mean tarenflurbil concentration in the CSF was ∼1.2μM, some 200-fold below its EC_50_ concentration for the inhibition of Aβ42 production in cell culture. There was no lowering of Aβ42 levels in the CSF at any dose.

#### Did the Clinical Program Establish That the Drug Was Mediating the Desired Effect, and How Robust Were the Phase 2 Data That Were Used to Progress to a Phase 3 Trial?

The phase 2 clinical trial studied 210 mild–moderate AD patients with MMSE scores between 15 and 26.^46^ Patients received either tarenflurbil 400mg b.i.d. (n = 69), 800mg b.i.d. (n = 70), or placebo (n = 71) for 12 months in a multicenter, placebo-controlled double-blind study. The primary outcome measures were ADAS-cog and 1 functional assessment, either the Alzheimer's Disease Cooperative Study–Activities of Daily Living Inventory (ADCS-ADL) or the CDR-SB. An analysis showed an apparent interaction between the baseline cognitive and functional scores and treatment effect, so that efficacy analyses were performed separately for the mild (MMSE > 20) and moderate AD patients. When the analyses were performed in this way, 800mg b.i.d. tarenflurbil-treated patients showed a significantly slower rate of decline of ADCS-ADL; ADAS-cog and CDR-SB showed similar effect sizes but were not statistically significant. In the moderate AD group (MMSE ≤ 19), the placebo group demonstrated a significantly lower rate of decline in all 3 outcome measures than did the 800mg b.i.d. tarenflurbil group. These somewhat paradoxical findings are difficult to interpret. However, the change in ADCS-ADL over the 12-month period was higher in the mild AD placebo group than the moderate AD placebo group, whereas for the 2 other primary outcome measures, ADAS-cog and CDR-SB, the moderate AD group showed greater deterioration, as might be expected. Thus, the effect seen at 800mg b.i.d. tarenflurbil in mild AD patients might have been due to an unusually large placebo group deterioration rather than a bona fide treatment effect. Importantly, proof of mechanism—a change in the spectrum of Aβ metabolites in the CSF in favor of shorter forms—was not assessed.

#### Tarenflurbil Phase 3 Trial

The phase 3 study enrolled 1,646 mild AD patients in a multisite, randomized, double-blind placebo-controlled trial comparing 800mg b.i.d. tarenflurbil versus placebo for 18 months.^47^ The primary outcome measures were change at 18 months from baseline on ADAS-cog and ADCS-ADL. There was no difference between the drug-treated and placebo-treated groups on the primary outcome measures, and CSF analyses of Aβ metabolite spectrum were not performed.

### Semagacestat

#### What Was the Hypothesis Being Tested?

γ-Secretase activity is required to release the Aβ peptide,^48^,^49^ hence inhibitors of γ-secretase should reduce Aβ production. In the simplest interpretation of the amyloid hypothesis, which posits that continued deposition of Aβ drives pathological processes resulting in neuronal dysfunction and death, a γ-secretase inhibitor (GSI) that reduced Aβ production would slow the progression of AD. Semagacestat is a "classical" GSI,^50^ acting as a noncompetitive enzyme inhibitor with an allosteric binding site.

#### Did the Preclinical Data Offer Support for the Hypothesis?

γ-Secretase is responsible for the final cleavage of the APP C-terminal domain following cleavage by either α- or β-secretase and also cleaves a wide range of substrates, including Notch.^51^,^52^ The Notch signaling pathway is critical for cell fate determination in many dividing cells and is therefore a significant potential safety liability for a GSI. Several drug discovery programs have sought compounds that were selective for Aβ versus Notch inhibition so as to provide a margin of safety.^53^–^55^ However, the in vitro assays (cell-free and cell-based) employed, although they do allow compounds to be compared with each other, are of unknown predictive validity for the in vivo situation. Semagacestat inhibited Aβ production with EC_50_ = 14.9nM in HEK293 cells stably transfected with hAPPSwe cDNA.^56^ In HEK293 cells stably transfected with the Notch δE cDNA construct, semagacestat inhibited the production of the Notch intracellular domain with EC_50_ = 46nM (P. C. May, personal communication). This indicated that semagacestat has a cell-based Aβ inhibition/Notch inhibition ratio of ∼3. The dose-related inhibition of Aβ production in cell-based assays has been widely replicated but with slightly different potencies and consequently different Aβ/Notch inhibition ratios: for example, 1.3,^57^ 0.8,^55^ and 20.5.^58^ The most informative study was performed using a cell-free, quantitative γ-secretase in vitro assay where Notch and APP substrate concentrations were accurately controlled.^59^ This demonstrated an Aβ/Notch ratio of 0.1. These data suggest that for semagacestat, the separation of inhibition of Aβ production over Notch inhibition was marginal.

Preclinical in vitro and in vivo studies revealed that the pharmacology of semagacestat and of GSIs in general was complex. This led to a biphasic stimulation/inhibition of Aβ production determined by both substrate availability and compound concentration.^58^,^60^,^61^ The mechanistic explanation for this effect remains obscure. In vivo experiments demonstrated a similar stimulation/inhibition effect of semagacestat on plasma Aβ levels, but this was not demonstrated in mouse brain,^62^ guinea pig brain,^61^ or rat brain.^58^ Semagacestat was also orally administered at 2mg/kg acutely to beagle dogs to assess the pharmacokinetic and pharmacodynamic profile in plasma and in CSF.^63^ This study showed that Aβ40 and Aβ42 peptides were lowered in the CSF by up to 60% and that suppression of Aβ production was sustained for longer in the CSF than in the plasma compartment. With lower doses of semagacestat, or at longer time-points at which point compound concentrations are declining, there was an elevation of Aβ in plasma that was not seen in the CSF.^64^ These data can be rationalized as follows. At low GSI and substrate concentrations, γ-secretase is stimulated. APP expression in peripheral tissues is lower than in the brain, hence peripherally derived Aβ is initially suppressed following an oral dose (when compound levels are high), but then stimulated as compound levels diminish. In the brain, where APP expression is higher, the stimulation of Aβ is less apparent. As Aβ is trafficked out of the brain rapidly,^65^ it might also be technically challenging to detect GSI-induced increases in Aβ levels.

In PDAPP transgenic mice, which overexpress the hAPP717 mutation,^66^ dose-related inhibition of brain Aβ production was demonstrated after acute and 7-day dosing.^67^ In a chronic study, semagacestat was administered daily to 5-month-old PDAPP mice for 5 months at 3, 10, and 30mg/kg.^68^ This resulted in dose-related reduction in insoluble brain Aβ that was significantly different from control groups at the highest dose for both Aβ40 and Aβ42. There was no significant reduction in plaque as measured immunohistochemically. Interestingly, semagacestat was a more potent inhibitor of Aβ40 than Aβ42 production, an effect seen by others using semagacestat^61^ and other GSIs of this class.^69^ Importantly, the dosing of semagacestat was initiated prior to the onset of Aβ plaque deposition in the PDAPP mice, and thus reflects a preventative rather than a therapeutic dosing paradigm. This is an important concept from 2 perspectives: first, in how it relates to its proposed clinical use; and second, because a therapeutic agent can inhibit Aβ deposition via fundamentally different mechanisms (see Fig 1). Several studies have investigated this issue and demonstrated that GSIs prevent the formation of new Aβ plaques, but even with significant suppression of Aβ production, do not mediate the clearance of existing plaques.^69^–^72^

### Preclinical to Clinical Translation

In healthy volunteers, semagacestat has a time to reach maximum concentration in plasma of 1 to 1.5 hours and a plasma half-life of 2.5 hours when administered daily for 14 days at doses ranging from 5 to 50mg/person. There was a dose-related reduction in plasma Aβ, followed by a stimulation of up to 500% over baseline for the lowest dose of semagacestat.^73^ In this study, no reduction in CSF Aβ could be detected when sampled 6 hours after compound dosing. In a phase 2 study, semagacestat was given at 30mg every day (q.d.) for 1 week followed by 40 mg q.d. for 5 weeks to 33 mild–moderate AD patients.^74^ At the end of the study, there was evidence of Notch-related effects on lymphocytes, but on the whole the drug was well tolerated. There was a 38% suppression of plasma Aβ40 but no effect on CSF Aβ40/42.

In the preclinical studies in the PDAPP mouse, 30mg/kg given once daily for 5 months reduced deposited Aβ and suppressed plasma Aβ by approximately 60% at maximal drug concentration. Thus, this level of plasma Aβ reduction was sought in human studies as a translational biomarker. Accordingly, a phase 1 study investigated the Aβ pharmacodynamic effect of 3 doses of semagacestat: 60, 100, or 140mg in normal humans.^75^ Blood samples were taken at regular intervals up to 24 hours postdose for analysis of compound, and Aβ concentration and CSF samples were collected 4 hours after dosing. The maximum percentage decrease in plasma Aβ from baseline values was 50% for the 60mg group and 73% for the 140mg dose, and occurred between 4 and 6 hours postdose, returning to baseline values between 8 and 13 hours later, depending on the dose. There were slight reductions in CSF Aβ that were significant for Aβ40 at the 140mg dose. As seen previously, there was a large increase in plasma Aβ that followed the initial suppression phase.

Although the plasma biomarker response confirmed that γ-secretase was being inhibited in a dose-related manner, there was no evidence that the production of brain Aβ was being affected. Given the excellent brain penetrant properties of semagacestat, it was unlikely that brain γ-secretase was unaffected by the compound, and the most likely explanation for the lack of a measurable Aβ response lay in the technical challenge of measuring CSF Aβ.

#### Did the Clinical Program Establish That the Drug Was Mediating the Desired Effect, and How Robust Were the Phase 2 Data That Were Used to Progress to a Phase 3 Trial?

The inhibition of brain Aβ production by semagacestat was measured using the stable isotope kinetic effect assay.^65^ Humans were given a continuous intravenous (i.v.) infusion of ^13^C-leucine for 9 hours to isotope-label proteins. CSF was collected via a spinal tap every hour for up to 36 hours, and Aβ species were immunoprecipitated using a mid-domain antibody before mass spectrometry analysis. The fractional incorporation of ^13^C-leucine was used to analyze the rate of production and clearance of Aβ. Semagacestat was administered in a single oral dose of 100, 140, and 280mg, and the effects on brain Aβ synthesis and clearance were measured.^76^ This crucial study proved that semagacestat was able to inhibit brain Aβ production by 47%, 52%, and 84% at 100, 140, and 280mg doses, respectively, over a 12-hour period.

A phase 2 safety study^77^ investigated the tolerability of 100 and 140mg once daily dosing over a 12-week period in mild–moderate AD patients. Although the drug was well tolerated overall, there was an increased incidence of skin rashes and hair color changes, which were indicative of inhibition of Notch signaling. In retrospect, it is noteworthy that both doses numerically worsened ADAS-cog scores. Plasma Aβ levels were inhibited by 65% at the 140mg dose.

It is apparent that semagacestat was cautiously developed, and that given the side effect profile of Notch inhibition, it was not possible to increase the dose above 140mg q.d. to garner increased efficacy.

#### Semagacestat Phase 3 Trials

Two phase 3 trials (Identity 1 and Identity 2, ClinicalTrials.gov identifiers NCT00594568 and NTC0076241122600) planned to enroll 2,600 mild–moderate AD patients who were randomized to placebo, 100mg semagacestat, and 140mg semagacestat once daily for 76 weeks in 2 trials. The ADAS-cog and ADCS-ADL were the coprimary outcome measures. These trials were halted after an interim futility analysis of Identity 1 showed a significantly increased incidence of skin cancer, infections, and white blood cell and other hematologic abnormalities.^78^ There was no improvement in cognition as measured by the ADAS-cog, and activities of daily living were significantly worsened at the highest dose. The 140mg dose showed a significant worsening of the CDR-SB and the MMSE. CSF levels of Aβ40, Aβ42, and tau were not altered by semagacestat treatment, whereas phospho-tau 181 (ptau) was significantly, but modestly, reduced. There were no drug effects on fluorodeoxyglucose positron emission tomography (PET), ^18^F-florbetapir PET to measure deposited brain Aβ, or volumetric MRI. At the end of a 32-week safety extension phase, after cessation of dosing, there was no difference in the changes from baseline in the coprimary measures across the 3 groups; other abnormalities in immune and renal function had not fully resolved.

It is likely, given that γ-secretase has many substrates, that the deleterious effects mediated by semagacestat are unrelated to Aβ metabolism but will never be elucidated.

### Bapineuzumab

#### What Was the Hypothesis Being Tested?

The hypothesis being tested was that administration of an antibody directed at the N-terminus of Aβ would mediate the clearance of Aβ plaque from the brain parenchyma of AD patients and thereby reduce the progression of cognitive decline and deterioration of activities of daily living in AD.

#### Did the Preclinical Data Offer Support for the Hypothesis?

The development of bapineuzumab stems from a groundbreaking study showing that immunization of PDAPP transgenic mice with Aβ42 peptide was able to prevent the deposition of Aβ plaque in the brain parenchyma.^79^ This seminal work opened up the potential for using antibodies as therapeutic agents for AD, which although considered earlier^80^ had been largely discounted owing to their poor blood–brain barrier (BBB) penetration. The original study was followed by others that confirmed the general principle that immunization with Aβ42 was able to produce anti-Aβ antibodies that acted to prevent Aβ plaque deposition.^81^,^82^ The immunization protocol effectively prevented the brain accumulation of insoluble Aβ when antibodies were raised prior to the period of Aβ deposition. This work led to the clinical development of AN1792, an active immunization using Aβ42 peptide as the immunogen. AN1792 was halted during its phase 2 study due to an unacceptable incidence (6%) of meningoencephalitis,^83^ likely due to the addition of polysorbate 80 to the immunization formulation that resulted in an inflammatory Th1-cell–mediated response.^84^ Several studies have investigated the consequences of Aβ vaccination in some patients that have since died, and their brains have been made available for postmortem neuropathology. These publications have been informative, but caution must be exercised, because the group sizes are low, the appropriate controls (patients receiving placebo) have not been available, and certain findings that were statistically significant failed to replicate when the cohort studied was enlarged.^85^,^86^ Also, the same patients are analyzed in multiple publications.^87^ Several studies revealed that AN1792 immunization appeared to reduce parenchymal Aβ plaque but without affecting tau pathology^88^–^92^ (total number of immunized patients = 13). Other authors have reported that tau pathology was modestly reduced and that neurite morphology was normalized in Aβ-immunized patients^87^ (number of immunized patients = 5). Also, 1 study demonstrated that whereas parenchymal plaque was reduced, total soluble amyloid levels were increased in gray and white matter^93^ (number of immunized patients = 2). In terms of the inflammatory status of the brain, it was surprisingly shown that overall microglial activation was lowered in AN1792-immunized patients^86^ (number of immunized patients = 11). In summary, these tantalizing studies offer some evidence (albeit without appropriate controls) for antibody-mediated resolution of deposited Aβ, and reinforce the value of having brain donation as an important component of AD clinical trials. The AN1792 antibody response was predominantly to the free N-terminus of Aβ^94^; hence, this epitope was targeted with a passive immunization approach. Bapineuzumab is the humanized version of 3D6, a highly specific mouse monoclonal antibody raised to the Aβ amino acid residues 1–5.^95^ 3D6 is a mouse IgG2b antibody with an affinity for soluble Aβ of <30nM^96^ and between 3 and 5nM.^97^ The dissociation constant (K_d_) of the bapineuzumab Fab fragment for Aβ1–40 was recently reported to be 89nM.^98^ When 11.5-month-old PDAPP mice were treated with weekly injections of 3D6 for 6 months, the Aβ burden (percentage of a brain section of the frontal cortex that can be immunohistochemically stained for Aβ) was reduced by 86%.^96^ However, it is unclear whether this regime reduced Aβ levels to below those present at the start of dosing, Aβ was not quantitated using biochemical assays, and the potency of 3D6 cannot be calculated, because the dose administered was not reported. In a second study, 3D6 was administered (dose not reported) to 13-month-old PDAPP mice for either 3 or 35 days. In this experiment, although there was some semiquantitative evidence for a reduction in small and diffuse plaques, there was no quantitative data on insoluble Aβ levels, and so the overall efficacy of the treatment is unknown. The mechanism by which anti-Aβ antibodies clear brain Aβ was investigated using an ex vivo assay in which 3D6 or 3D6 Fab fragments were administered together with mouse microglia cells onto nonfixed brain slices taken from either AD brains or PDAPP mouse brains. In this assay system, the immunohistochemical analysis suggested that Fc-mediated microglial phagocytosis was a likely mechanism by which Aβ plaques could be removed from the brain. Thus, the therapeutic hypothesis was that passive immunization with bapineuzumab would remove Aβ plaques from the brains of AD patients, most probably via stimulation of microglial activation, but also potentially via direct mechanical disruption of Aβ plaque mediated by antibody binding^99^ and the direct inhibition of Aβ fibrillogenesis.^100^ An in vivo study that involved the direct application of antibody to the brain via a cranial window provided evidence that both intact and Fab fragments of 3D6 were able to mediate resolution of plaque in a 3-day time period as measured by multiphoton imaging.^99^ The translatability of this work to systemic dosing is challenging, because there was evidence that the opening of the skull caused an inflammatory response, and the concentrations of antibodies administered were not reported. The preclinical data therefore supported the concept that passive immunization of bapineuzumab in AD patients would result in a slowing of Aβ deposition, but there were no quantitative data to support a reduction of Aβ plaque below that present at the start of dosing. A study was performed to establish target engagement that entailed systemic injection of trace amounts of ^125^I-3D6 into PDAPP transgenic mice at various ages.^101^ Radioactivity was higher in the hippocampus than in the cerebellum, and radioactivity in the hippocampus increased from day 7 to day 14 after ^125^I-3D6 injection. Surprisingly, there was very little difference in binding between the cortex and the cerebellum, although it is known that the cortex develops Aβ plaques in the PDAPP model whereas the cerebellum does not.^95^ In a competition experiment, up to 30mg/kg of unlabeled 3D6 was unable to compete with up to 170ng/mouse of ^125^I-3D6 binding that was measured in cerebellum, hippocampus, or cortex. Although this could mean that antibody binding sites on deposited Aβ could not be saturated, this protocol is similar to a classic competition experiment that is used to determine specific from nonspecific binding. Therefore, a different interpretation is that a significant proportion of the ^125^I-3D6 binding is nonspecific. In a different study,^97^ it was demonstrated in 24- to 29-month-old PDAPP mice that 40mg/kg of biotinylated 3D6 was unable to bind to deposited Aβ plaque. The authors concluded that the antibody is bound by soluble Aβ that might be present in increased concentration around plaques as a consequence of insoluble-to-soluble phase Aβ exchange. If this is true, then 3D6 would be unable to access deposited plaque to mediate microglial-mediated Aβ clearance.

In another study, 50mg/kg intraperitoneally (i.p.) per week of 3D6 administered for 6 weeks resulted in a significant increase in the severity and incidence of microhemorrhage.^102^ In a later study, using younger 12-month-old mice and much lower doses of 3D6 (loading dose of 7.5mg/kg, followed by maintenance dose of 3mg/kg; loading dose of 0.75mg/kg, followed by maintenance dose of 0.3mg/kg; loading dose of 0.25mg/kg, followed by maintenance dose of 0.1mg/kg; treatments administered weekly for 6 months), it was demonstrated that although 3D6 increased the incidence of microhemorrhage, this could be ameliorated at lower doses.^103^ In addition, it was demonstrated that 3D6 treatment removed vascular amyloid and potentially prevented deposition. At the start of the study, the incidence of vascular amyloid deposits in PDAPP mice was ∼40%, thus it is not possible to differentiate between the effects of 3D6 to prevent vascular amyloid deposition as opposed to 3D6 clearing existing vascular amyloid.

#### Preclinical to Clinical Translation

To date, 2 preclinical studies have been published where the dose of 3D6 is reported and where a reduction in plaque deposition was found. Seubert et al performed 3 studies in PDAPP transgenic mice.^104^ In a preventative study, 3D6 (IgG2a) was administered i.p. at 10mg/kg/wk to 4-month-old PDAPP mice for 12 months. 3D6 treatment dramatically reduced deposited Aβ total accrual by 89% measured using specific Aβ ELISAs. When 3D6 was administered in a therapeutic study to 12-month-old mice at 3mg/kg/wk for 6 months, there was a 93% reduction in immunohistochemical staining compared to controls. Aβ was not quantitated biochemically for this study, but in a second identical study Aβ total accrual was reduced by 77% and plaque load by 98%. In these studies, it is not possible to confirm whether 3D6 reduced Aβ deposition below predosing levels. Also, although a preclinical study can be readily established to be preventative because there is no deposited Aβ present at the start of the study, judging whether a study is therapeutic and moreover translatable to human disease is much more challenging, because it requires an assessment of how the deposited Aβ levels in the model system relate to the human disease. This point was investigated by Demattos et al^97^ in a series of experiments with 3D6 (IgG2b). At 5.5 months of age, PDAPP mice have not begun to deposit Aβ in brain parenchyma. 3D6 was administered to ∼5.5-month-old PDAPP mice for 7 months at a dose of 12.5mg/kg s.c. once weekly. This dosing regimen produced a highly significant 68% reduction in deposited Aβ42 accrual in the hippocampus. When administered at 12.5mg/kg once weekly s.c. for 3 months to PDAPP mice at a starting age of 9 months, although 3D6 was again able to reduce deposited Aβ42 statistically significantly compared to the control group, the treatment failed to reduce the levels of Aβ to below the levels present at the start of the study. When 3D6 was administered to mice at 18 months or 23.5 months of age, 3D6 failed to inhibit deposited Aβ42 when compared to the control group. At these 2 time periods, Aβ accrual in control animals has reached a plateau, and in this respect resembles AD. However, in the latter study it was demonstrated that 3D6 mediated a significant increase in microhemorrhage despite the lack of efficacy on parenchymal plaque removal. In summary, the preclinical data support a profile where 3D6 is robustly efficacious in a preventative rather than in a therapeutic dosing paradigm. However, one must be cautious in extrapolating from a transgenic model that has rapid Aβ deposition to man, where Aβ deposition takes place over many years.

#### Did the Clinical Program Establish That the Drug Was Mediating the Desired Effect, and How Robust Were the Phase 2 Data That Were Used to Progress to a Phase 3 Trial?

Bapineuzumab is the humanized IgG1 version of 3D6. In the phase 2 multiple ascending single-dose study, 0.5, 1.5, and 5.0mg/kg bapineuzumab were given to mild–moderate AD patients via i.v. infusion.^105^ This established the mean half-life of bapineuzumab to be 23.7 days. Vasogenic edema (now referred to as amyloid-related imaging abnormality–edema [ARIA-E]) was identified in 3 of 10 patients receiving the 5mg/kg dose, with 1 of these patients exhibiting microhemorrhage (now referred to as ARIA-M). Based on these data, a phase 2 study enrolled 234 mild–moderate AD patients who were assigned to receive 0.15, 0.5, 1.0, or 2.0mg/kg bapineuzumab, or a placebo given by i.v. infusion every 13 weeks for 78 weeks.^106^ This was powered as a safety study, but the coprimary efficacy endpoints were the ADAS-cog and Disability Assessment for Dementia (DAD). Other study assessments included the Neuropsychological Test Battery, CDR-SB, and exploratory CSF and imaging biomarkers. There were no significant differences in the ADAS-cog and DAD between placebo and any of the bapineuzumab dose groups. An exploratory analysis was conducted on those patients who had received all 6 bapineuzumab infusions. When all bapineuzumab dose cohorts were pooled, there was a significant difference at week 78 between bapineuzumab-treated patients and placebo on the ADAS-cog and DAD measurements. The incidence of ARIA-E increased with increasing bapineuzumab dose, with the highest 2.0mg/kg dose resulting in a 27% incidence. ARIA-E also increased with *APOE4* gene dosage, with 2 copies of the gene resulting in a 33.3% incidence in bapineuzumab-treated patients. There were no episodes of ARIA-E in the placebo group. Using an identical dosing regimen in mild–moderate AD patients, the effects of bapineuzumab on ^11^C-Pittsburgh compound B (PiB) PET imaging were investigated.^107^ Patients were given ^11^C-PiB PET scans at baseline and then at weeks 20, 45, and 78. In bapineuzumab-treated patients, there was an 8.5% decrease in PiB retention compared with a 16.9% increase in the placebo group, and this difference was statistically significant. The number of patients in this trial was low (7 in the placebo group and 19 in the bapineuzumab-treated group), but nevertheless this finding was consistent with the proposed mechanism of action. CSF samples were taken from some of the patients in these 2 phase 2 trials, and Aβx–42, Aβ1–42, total tau, and ptau were measured. These samples were pooled to give 27 bapineuzumab and 19 placebo samples, and revealed that there was a significant reduction in ptau, but no effect on the other metabolites,^108^ an effect consistent with a reduction in disease-related neuronal loss or damage.

These phase 2 data provided some evidence that bapineuzumab treatment may be disease modifying. There were data suggesting a reduction in amyloid plaque and a decrease in CSF ptau, and there was some evidence of cognitive benefit. It was also clear that ARIA-E and ARIA-M were significant treatment adverse events that were more common in *APOE4* gene carriers.

#### Bapineuzumab Phase 3 Trial

Four phase 3 trials were launched involving a total of 4,570 mild–moderate AD patients randomized to placebo and bapineuzumab.^109^ Two separate trials were conducted in *APOE4* carriers and noncarriers predominantly in North America (ClinicalTrials.gov identifiers NCT00575055 and NCT00574132); 2 parallel trials were conducted predominantly in the rest of the world (NCT00667810 and NCT00676143). Bapineuzumab was administered to patients who were *APOE4* carriers at 0.5mg/kg and in *APOE4* noncarriers at 0.5mg/kg, 1mg/kg, and 2mg/kg initially, with the 2mg/kg dose being abandoned due to ARIA-E and ARIA-M. Bapineuzumab failed to meet the primary outcome measures on ADAS-cog and DAD in trials NCT00575055 and NCT00574132. Consequently, trials NCT00667810 and NCT00676143 were terminated. Of note, although the group sizes were low, there was some evidence in *APOE4* carriers of a modest reduction in PiB PET binding when compared with the placebo group, although in contrast to the phase 2 study, there was no difference when compared with the baseline values. There was also a statistically significant although modest reduction in ptau in both the *APOE4* carriers and noncarriers. On pooled data from trials NCT00575055 and NCT00574132, there was a significant but modest dose-related increase in brain atrophy due to bapineuzumab therapy. As was seen in the phase 2 studies, there was a dose-related increase in ARIA-E with bapineuzumab therapy that was more prevalent in *APOE4* carriers (21% at the 0.5mg/kg dose, and 6% and 13% at the 0.5mg/kg and 1.0mg/kg doses, respectively) than in *APOE4* noncarriers. Given the lack of clinical benefit, the biological significance of these biomarker changes is difficult to interpret.

### Solanezumab

#### What Was the Hypothesis Being Tested?

Solanezumab is a humanized IgG1 antibody derived from the mouse monoclonal antibody m266. The m266 monoclonal antibody was raised to a peptide-conjugate containing Aβ13–28.^110^ The antibody m266 recognizes an epitope in the Aβ16–24 midregion^111^ with low picomolar affinity.^112^ Solanezumab recognizes soluble, monomeric Aβ, but not deposited Aβ or amyloid plaques.^102^,^112^ In in vitro studies, m266 was able to deplete solutions of Aβ mixed with APOE, bovine serum albumin, or mouse IgG when separated by a 25kDa cutoff dialysis membrane, effectively acting as an Aβ "sink."^112^ Further experiments in PDAPP transgenic mice demonstrated that m266 was able to capture Aβ40 and Aβ42 in the plasma, such that a 0.5mg i.v. treatment with m266 resulted in the total capture of plasma Aβ. If human Aβ was injected into the cisterna magna of nontransgenic mice, then this could be subsequently detected bound to m266 in mice pretreated with the antibody, thus demonstrating the ability of m266 to capture Aβ effluxed from the central nervous system (CNS). In further experiments, it was demonstrated that although CSF and peripheral concentrations of Aβ were positively correlated in PDAPP mice lacking deposited Aβ, this correlation was lost when deposited Aβ was present in the brain.^113^ However, when 0.5mg m266 was administered i.v. to PDAPP mice, a positive correlation was demonstrated between hippocampal amyloid and the total amount of plasma Aβ measured at 24 hours. This result established that peripherally administered m266 was able to sequester Aβ and act as a peripheral sink for Aβ effluxed from the brain. Thus, the hypothesis for solanezumab treatment was that by administering the humanized version of m266 to AD patients, the net efflux of Aβ from the brains of AD patients would be augmented, leading ultimately to resolution or a decrease of deposited Aβ. This hypothesis rests on the assumption that deposited Aβ and soluble Aβ in interstitial CSF are in equilibrium.

#### Did the Preclinical Data Offer Support for the Hypothesis?

Whereas the data supporting the peripheral sink effects of m266 are robust, data showing that m266 treatment will reduce deposited parenchymal Aβ were not compelling. The antibody m266 was administered at 0.5mg/mouse i.p. every 2 weeks for 5 months in 4-month-old PDAPP mice, likely prior to the deposition of Aβ. At 9 months, the number of mice with <50% of the cortex immunohistochemistry for Aβ was reduced in m266-treated mice. However, insoluble total Aβ and Aβ42 were not significantly different from control.^112^ Furthermore, this experiment represents a preventative rather than a therapeutic treatment paradigm. Seubert and colleagues administered m266 at 10 and 3mg/kg/wk i.p. in 2 separate studies.^104^ Dosing was started at 12 months and continued until 18 months. At the 10mg/kg dose, m266 failed to reduce deposited Aβ as measured using immunohistochemistry; there was a trend for increased amyloidosis. At the 3mg/kg dose, m266 failed to show efficacy as measured either by Aβ immunohistochemistry or by quantitative ELISA of total brain Aβ. In a preventative dosing regimen, where 10mg/kg/wk m266 was administered from 4 to 16 months of age, again m266 failed to demonstrate efficacy as assessed either by Aβ immunohistochemistry or quantitative ELISA.

#### Preclinical to Clinical Translation

Solanezumab, the humanized version of m266, was given to AD patients in single-dose, dose escalation study in a total of 19 patients.^114^ The primary outcome measure was to assess safety, with a secondary outcome being pharmacokinetic and pharmacodynamic measurements. Solanezumab was administered at 0.5, 1.5, 4, and 10mg/kg i.v. in a saline infusion protocol. All doses were well tolerated, with no evidence for ARIA-E or ARIA-M. Large increases in plasma Aβ40 and Aβ42 were measured in a dose-related fashion. Although the analyses were not able to distinguish between free and antibody-bound Aβ, the sustained increase in plasma Aβ suggests that plasma Aβ was being captured for up to 42 days postdose. Interestingly, there was a dose-related increase in CSF Aβ as well, which was likely mediated by capture of Aβ by the 0.1% of the peripheral concentration of solanezumab that crossed the BBB. There were no effects on cognitive measures.

#### Did the Clinical Program Establish That the Drug Was Mediating the Desired Effect, and How Robust Were the Phase 2 Data That Were Used to Progress to a Phase 3 Trial?

The phase 2 program studied 52 AD patients in a placebo-controlled, randomized trial. Several dosing regimens were compared over a 12-week period: 100mg solanezumab every 4 weeks, 100mg weekly, 400mg every 4 weeks, 400 mg weekly. The primary outcome measure was the safety and tolerability of multiple administrations of solanezumab, with pharmacokinetic and cognitive assessments as secondary endpoints. There was a rapid, dose-related and dose regimen–related increase in plasma Aβ40 and Aβ42. Treatment-emergent adverse events were not different between solanezumab-treated patients and placebo controls. There was no evidence of ARIA-E or ARIA-M, or of meningoencephalitis, and no treatment effects measureable by ADAS-cog. In this study, antibody-bound and free Aβ40 and Aβ42 were assayed in CSF samples. These data showed a dose-related and dose regimen–related increase in total (bound plus unbound) Aβ40 and Aβ42 compared to baseline values. For unbound Aβ, there was no treatment effect on Aβ40, but for Aβ42 there was a dose-related and dose regimen–related increase. The increase in total Aβ40 (bound and unbound) is most likely due to capture by solanezumab entering the CNS. The increase in Aβ42 (bound and unbound), although somewhat counterintuitive, might herald some dissolution of amyloid plaques that are predominantly comprised of Aβ1–42. Thus, the mechanism of action of solanezumab was demonstrated in the phase 2 trial with circumstantial evidence of an effect on Aβ plaque.

#### Solanezumab Phase 3 Trials

Solanezumab was tested in 2 randomized, blinded, placebo-controlled phase 3 trials, Expedition 1 (1,000 mild–moderate AD patients) and Expedition 2 (1,040 mild–moderate AD patients; ClinicalTrials.gov identifiers NCT00905372 and NCT00904683).^115^ Solanezumab was administered via i.v. infusion at 400mg per patient every 4 weeks for 80 weeks. The coprimary outcome measures for both trials were improvement on change from baseline to week 80 in ADAS-cog and ADCS-ADL. Secondary outcome measures included volumetric MRI, CSF ptau, tau, CSF Aβ, Amyvid PET amyloid imaging, and plasma Aβ. Expedition 1 failed to reach its coprimary outcomes.^115^ However, in a prespecified secondary analysis in mild AD patients (MMSE = 20–26), solanezumab significantly improved cognitive performance as measured by ADAS-cog11 and the ADAS-cog14 scale, which is more sensitive to changes in mild-AD, but failed to demonstrate an improvement in activities of daily living. As the data from Expedition 1 were available prior to the termination of Expedition 2, the primary outcome measures for Expedition 2 were changed to a single outcome of improvement in ADAS-cog14 in the mild AD patient cohort (MMSE = 20–26) measured at 80 weeks. Solanezumab failed to meet its primary outcome measure in Expedition 2. When data from both trials were pooled, then solanezumab therapy significantly improved cognitive performance as measured by ADAS-cog14, but this improvement was driven largely from the Expedition 1 result. The pooled data in the mild AD cohort failed to reveal an improvement in ADCS-ADL, although there was a positive trend. Of note, in both the bapineuzumab and solanezumab phase 3 trials, amyloid PET imaging suggested that approximately 25% of the mild AD cohort did not have amyloid deposits and thus could not respond to amyloidocentric therapeutic agents.^109^,^115^,^116^ Solanezumab did not produce significant effects on deposited amyloid as measured with Amyvid PET amyloid imaging agent. As was seen in the phase 2 studies, there was a significant increase in total (antibody-bound and free) CSF Aβ42 and Aβ40 and a reduction in free CSF Aβ40, but no increase in free CSF Aβ42. As previously, there was a very large increase in plasma Aβ bound to antibody. There were no solanezumab-mediated changes in ptau levels in the CSF and no change in hippocampal or whole brain volume as measured by volumetric MRI as a consequence of therapy. However, in those patients who were treated with solanezumab and who were amyloid positive as assessed by Amyvid PET, there was a nonsignificant increase in atrophy.

Based on the data from Expedition 1 and 2, solanezumab is currently being tested in Expedition 3, a phase 3 trial in mild AD (ClinicalTrials.gov identifier NCT01900665) with improvement in ADAS-cog14 and ADCS-ADL as coprimary endpoints and a positive amyloid PET brain scan as an inclusion criterion. The duration and dosing regimen for Expedition 3 are identical to those for Expedition 1 and 2.

### Intravenous Immunoglobulin G

#### What Was the Hypothesis Being Tested?

Intravenous immunoglobulin G (IVIg) is a preparation of pooled polyspecific IgG obtained from the plasma of large numbers of healthy individuals. IVIg is used to treat immunodeficiency and inflammatory syndromes. By using Epstein–Barr virus to immortalize B cells taken from AD patients,^117^ it was demonstrated that anti-Aβ antibodies could be detected that recognized the N-terminus of the Aβ peptide but that were also conformational. It was further demonstrated that about 10-fold more B-cell lines immortalized from AD patients were producing anti-Aβ antibodies than from controls.^118^ In contrast, later studies^119^ demonstrated that anti-Aβ antibodies could be detected in CSF and blood in AD patients, but at lower titers than in controls. Further investigations demonstrated that several commercial IVIg preparations also contained anti-Aβ antibodies.^120^ When administered to 7 elderly patients with a variety of conditions, but not AD, the treatment reduced total Aβ and Aβ42 in CSF by approximately 20%. A retrospective analysis^121^ analyzed medical claims for patients >65 years of age from a national database and compared the incidence of AD over a 5-year period following IVIg. This revealed a 42% reduction in the risk of being diagnosed with AD following IVIg therapy. Together with the preclinical data^79^,^112^ demonstrating several potential therapeutic modalities for anti-Aβ antibodies, it was considered worthwhile to test the hypothesis that IVIg would elicit a therapeutic effect in AD patients.

#### Did the Preclinical Data Offer Support for the Hypothesis?

Given the nature and provenance of the therapy, there are few published preclinical experiments. Anti-Aβ antibodies affinity-purified from IVIg were shown to inhibit Aβ40 and Aβ42 fibril formation.^122^,^123^ The same purified preparations were also shown to inhibit Aβ-mediated toxicity in fetal rat primary hippocampal cells, but the interpretation of direct Aβ-mediated toxicity assays is challenging.^9^ Other workers also demonstrated an antiaggregatory effect of affinity-purified anti-Aβ antibodies on Aβ-induced toxicity, but no control antibody was used, and so the specificity of action is unclear.^124^ In other experiments, affinity-purified anti-Aβ antibodies at a high concentration of 20μM increased microglial-mediated Aβ clearance in an ex vivo assay using Aβ plaque–laden transgenic mouse brain slices. In APP23 mice, biodistribution of ^111^In-labeled affinity-purified anti-Aβ antibodies from IVIg was measured and compared with rituximab (as a control antibody), and the anti-Aβ mouse monoclonal antibodies 4G8 and 6E10.^125^ There was no significant difference in the brain binding between any of the antibodies at time periods up to 4 days, but interpretation is problematic, because it was unclear whether the APP23 mice used had deposited parenchymal Aβ. IVIg was administered i.p. at a dose of 1g/kg weekly to APP/PS1 transgenic mice with deposited parenchymal plaque for up to 14 weeks.^124^ Antihuman IgG antibodies were used to detect IVIg, and there was evidence for brain binding, but the experiment did not reveal specific binding to Aβ plaques.

#### Did the Clinical Program Establish That the Drug Was Mediating the Desired Effect, and How Robust Were the Phase 2 Data That Were Used to Progress to a Phase 3 Trial?

The development of IVIg was largely driven from human experimental medicine, and so the normal evolution of preclinical science to inform human dose setting and pharmacology was not followed.

The first description of a clinical study with IVIg was in a 1998–2000 study involving 8 AD patients treated for 12 months with a monthly dose of IVIg of 0.2g/kg. This study reported a significant improvement in patients but was never published as a peer-reviewed paper.^126^ In another study, 5 AD patients were given 0.4g/kg IVIg every month for 6 months in a non–placebo-controlled open study.^127^ Comparing baseline with measurements taken at the end of the study revealed a modest reduction in total Aβ in CSF, with no change in free Aβ42. There was an increase in total Aβ in serum (∼2.3-fold), which is marginal compared with the increases in serum Aβ mediated by solanezumab treatment (∼25,000-fold). The treated patients did not show a clinical decline over the 6-month period as measured by ADAS-cog and the MMSE. An open-label dose-ranging study was performed in 8 mild AD patients using an interrupted dosing design.^128^ Following a single dose of 0.4g/kg of IVIg, patients were randomly assigned to 0.4g/kg/2 weeks, 0.4g/kg/wk, 1g/kg/2 weeks, or 2g/kg/4 weeks for 6 months of treatment. IVIg was then withdrawn for 3 months, after which all patients were treated with 1g IVIg/kg/2 weeks for 3 months followed by 0.4g/kg/2 weeks for a further 6 months. Given that there were only 2 patients per dosing arm in this uncontrolled study, it is difficult to infer very much from the cognitive measures that were made. However, the data show a favorable change in MMSE over the first 6 months of dosing followed by a decline during the 3-month treatment withdrawal period. Data from a single patient on the highest dose of 2g/kg/4 weeks demonstrated increases in plasma Aβ following each administration, but these were minor (∼1.8-fold) compared with that achieved by solanezumab.

A randomized, phase 2 dose-finding trial was conducted in 55 mild–moderate AD patients with median area under curve (AUC) of plasma Aβ40 concentration taken between the last IVIg infusion and the final visit as the primary outcome measure.^129^ Six groups of between 6 and 8 patients were administered 0.2, 0.5, or 0.8g/kg IVIg every 4 weeks, or half these doses administered every 2 weeks, for 24 weeks. In this study, there was no significant increase in Aβ40 AUC following IVIg treatment at any dose; there was a significant decrease in the 0.4g/kg/2 weeks group.

An unpublished 6-month, double-blind, placebo-controlled study in 24 mild–moderate AD patients reported an improvement in the clinician global assessment scale in treated patients. Patients were entered into a 3-year open-label extension, and 4 patients who had received 0.4g/kg/2 weeks showed no further decline over this period.

In summary, the preclinical data did not offer much support for therapeutic efficacy for IVIg, although the nature of the drug made this a challenging prospect. The open-label phase 2 data gave a suggestion of a very small, acute increase in plasma Aβ following IVIg treatment. The randomized, placebo-controlled phase 2 studies, again in small numbers of patients, did not reveal the expected biomarker response.

#### Gammagard Phase 3 Trial

Gammagard IVIg was tested in the Gammaglobulin Alzheimer's Partnership 160701 study, a multicenter, randomized, placebo-controlled phase 3 trial in 390 mild–moderate AD patients (ClinicalTrials.gov identifier NCT00818662). The trial examined 2 doses of 0.2g/kg/2 weeks and 0.4g/kg/2weeks versus placebo for 18 months. IVIg failed to reach its coprimary outcomes, which were the 18-month change from baseline on the ADAS-cog and ADCS-ADL. This study is currently unpublished, and there has been no confirmation of whether large increases in plasma Aβ as a consequence of antibody capture, as was demonstrated for solanezumab as a biomarker response, were seen with IVIg.

## Conclusion

The amyloid hypothesis has significantly influenced drug discovery and development in AD over the past 20 years,^130^ but no amyloidocentric therapeutic agent has reached its primary outcome measures. This has led some in the field to question its validity. We are yet to understand the role (if any) of deposited amyloid, or other Aβ species, in AD. Does Aβ act to trigger a disease that becomes Aβ independent, is some threshold level of brain Aβ elevation required, or does Aβ drive pathological processes in a continuous fashion at all stages of the disease? Without insight into these fundamental questions, it is difficult to determine what constitutes a clinical test of the amyloid hypothesis. Our view is that if a therapeutic agent was able to prevent or significantly delay Aβ deposition without affecting the incidence of dementia, then the hypothesis as we understand it would be invalid. We do not discount the possibility that significantly reducing levels of Aβ in the context of pre-existing deposition may be beneficial; the therapies that we have reviewed have not achieved this level of efficacy.

**Figure 2:**
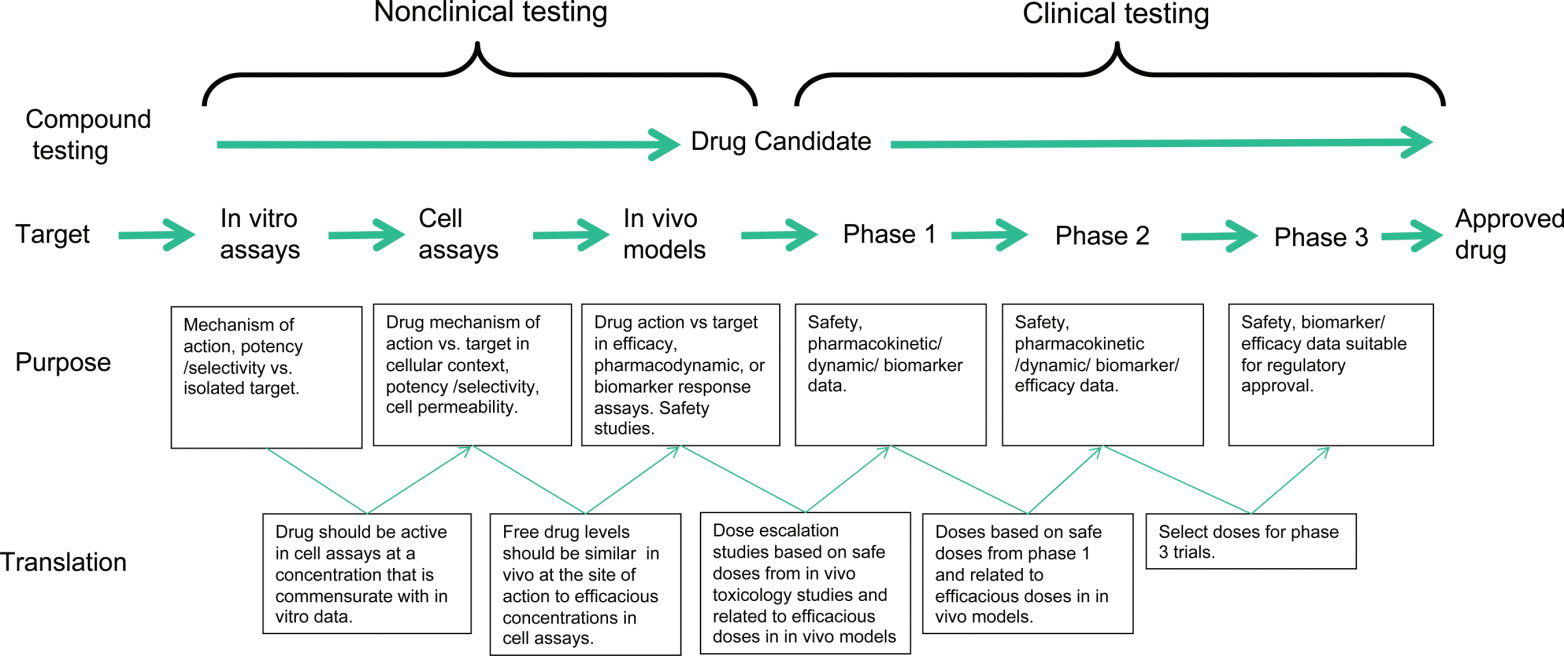
Many drug discovery programs progress through a logical sequence where the findings from one type of experiment inform the next step. Significant confidence is generated in programs where the data generated within each phase are concordant with subsequent phases. Programs that lack this translational quality are subject to increasing risk of failure. Drug Candidate is a therapeutic drug approach with sufficient safety and efficacy data to be administered to man.

**Figure 3:**
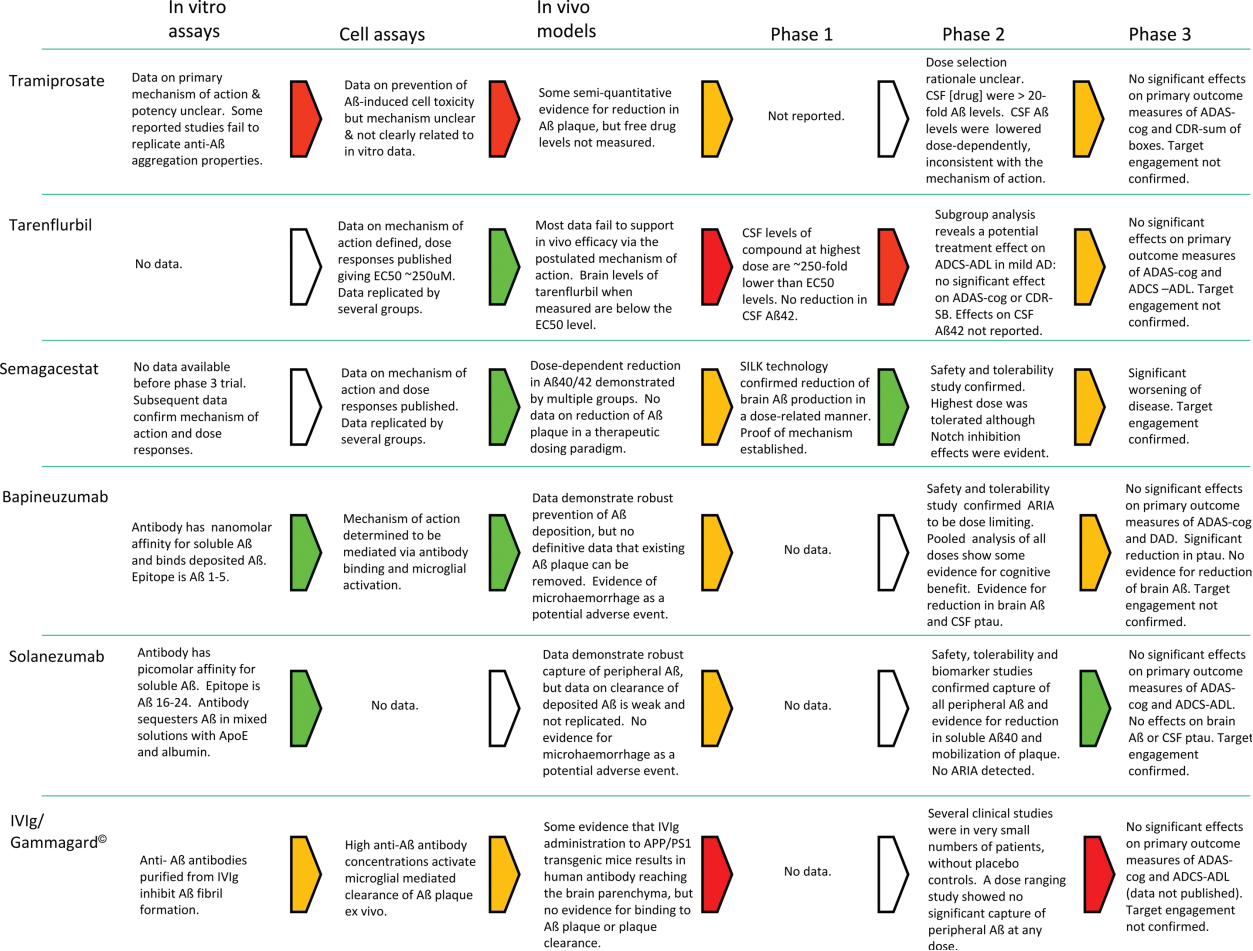
The key findings at each phase of the drug discovery process are summarized. These data must be seen in the context that all drug discovery programs, even those where each phase translates robustly into the following phase, are risky. Considerable judgement must be used during the program: for example, the interpretation of efficacy findings in transgenic mice and how well these may, or may not, translate to humans. Key: green = robust data support progression to next step; yellow = incomplete/inconsistent data indicate that progression involves significant risk; red = available data do not support progression; white = no data/not applicable. AD = Alzheimer disease; ADAS-cog = Alzheimer's Disease Assessment Scale–Cognitive Subscale; ADCS-ADL = Alzheimer's Disease Cooperative Study–Activities of Daily Living Inventory; APP = amyloid precursor protein; ARIA = amyloid-related imaging abnormality; CDR-SB = Clinical Dementia Rating–Sum of Boxes; CSF = cerebrospinal fluid; DAD = Disability Assessment for Dementia; EC50 = median effective concentration; IVIg = intravenous immunoglobulin G; SILK = stable isotope kinetic effect.

In an ideal world, drug discovery can operate in a stepwise fashion from preclinical to clinical experiments (Fig 2). Data from each segment of the drug discovery process is used to predict and interpret outcomes at the subsequent phase; thus, each activity has a translational value for the next step. As this schema is target and drug dependent, aspects of it are not relevant for some of the therapeutic approaches that we have reviewed; but the principle of logical progression remains important. Figure 3 provides a guide for how robust the data were to support the compound progression for each of the therapeutic approaches discussed. In several cases, there were significant gaps in the data. The best outcome for a phase 3 trial is that the therapeutic agent provides meaningful clinical benefit with acceptable safety; the worst outcome is that the therapeutic agent has no clinical benefit (or worsens disease outcomes) but does not unequivocally test the mechanistic approach, because target engagement was not measured. For the majority of the therapeutic agents analyzed, target engagement was not established. For tramiprosate, data supporting the primary hypothesis of an antiaggregatory effect on Aβ were weak and not replicated by other laboratories. The in vivo data were also mixed, with a lack in 1 case of quantitative estimates of insoluble Aβ, and in another a very large dose of tramiprosate was administered to demonstrate a reduction in insoluble Aβ that also showed a reduction in soluble Aβ as well; this would not have been expected for an Aβ aggregation inhibitor. Furthermore, dose–response relationships were not established. The rationale for the choice of clinical doses is not clear, but nonetheless a reduction in CSF Aβ was demonstrated in the phase 2 studies, although for this mechanistic approach an increase in CSF Aβ would have been anticipated, unless the therapeutic agent was able simultaneously to reduce aggregation and increase clearance, for which mechanistic support is lacking. In the phase 3 study, unanticipated variance precluded a full analysis of the trial, although there was no evidence for clinical benefit, and proof of mechanism biomarkers (eg, CSF Aβ) are not available. In summary, given the preclinical data, it is not surprising that tramiprosate failed. For tarenflurbil, the preclinical in vitro data demonstrating that the compound acted as a γ-secretase modulator were robust, but the in vivo data demonstrating effects on brain Aβ levels in tg2576 transgenic mice were not convincing, and tarenflurbil did not penetrate the brain at a sufficient concentration to mediate its pharmacological effect, either in the experiments performed in tg2576 transgenic mice or in man. Given this, it is not surprising that tarenflurbil did not demonstrate efficacy in man, although it would have been informative to have measured the Aβ metabolite spectrum in the CSF of patients who received the drug to confirm whether the therapeutic mechanism of action—a decrease in the longer forms of Aβ—had been achieved. The preclinical and clinical development of semagacestat was robustly prosecuted, although it is notable that there were very few subchronic in vivo studies performed to investigate the effects of the compound in therapeutic and preventative dosing regimens. Target engagement was definitively established in man, although given the mechanism and attendant Notch inhibition it was clear that the compound's therapeutic window was limited. The unexpected worsening of AD in treated patients, although very unfortunate, has at least closed this avenue of therapeutic enquiry; in this sense, the Identity trials were highly informative. The development of Gammagard represented a repurposing of an existing therapeutic agent. The preclinical and clinical data supporting the phase 3 program were insubstantial, and it is not surprising that Gammagard failed to provide clinical efficacy. Bapineuzumab was perhaps the most disappointing of the current phase 3 failures. There were a wealth of excellent preclinical in vivo data on the compound, although from the analysis performed herein it is also clear that the question of whether bapineuzumab (or the mouse progenitor 3D6) was able to remove existing plaque in a therapeutic dosing paradigm was not demonstrated, with the balance of data suggesting that it could not. The doses used in the clinical program were limited by ARIA, and finally the compound failed to show efficacy. Given the uncertainty regarding target engagement, we believe that the therapeutic benefit of clearing deposited plaque from the brains of AD patients remains to be tested. Although the preclinical in vivo data demonstrating the peripheral sink effect for solanezumab were very robust, and the translation into clinical doses was congruent, the data supporting an effect on clearance of deposited Aβ were weak. These data were also confounded somewhat because m266 recognizes both human and mouse Aβ, but nevertheless there is no evidence from the Expedition trials that solanezumab has an effect on Aβ plaque as measured using amyloid PET imaging. Thus, the beneficial clinical effects in mild AD—if replicated in Expedition 3—are extremely important both in providing a new therapeutic agent for AD and also in providing mechanistic data on the disease process itself.^116^

With hindsight, it is easy to pick out the gaps in data or logic in the programs that we have reviewed, but some of the inconsistencies were known at the time. The field is desperate to find a disease-modifying therapeutic agent for AD, which provides a powerful motivation for drug developers to sustain drug development programs. Furthermore, it is clear that the in vivo models we have are imperfect and of unknown predictive value to human disease. However, it is surprising that given the massive costs of clinical studies, the preclinical data are often relatively sparse. It is important that the field learns from the history of the development of amyloidocentric therapeutic agents so as to increase our chances of success in the future. In particular, drug developers should be encouraged to make all clinical data rapidly available so as to better inform the field. From our analysis, some of these lessons are quite simple: ensure full dose responses are measured in in vitro and in vivo systems; check that dose levels from isolated target, to cell-based assays, to in vivo experiments, are all sensibly translated; measure the therapeutic mechanism of action and target engagement in humans as early as possible; ensure that biomarker changes are congruent with the therapeutic mechanism of action; and do not change the therapeutic hypothesis mid-development. It also has to be recognized that chronic dosing experiments in mice can prove challenging because of the rapid metabolism of test agents, leading either to low overall exposures or to the requirement for more complex dosing regimens to sustain drug levels. Furthermore, simple replication of the preclinical experiments with appropriately powered group sizes and prespecified endpoints, in particular for in vivo experiments, would greatly enhance the quality of information needed to move therapeutic agents into—or out of—the clinical arena. Finally, an understanding of how the various transgenic mouse models of Aβ deposition relate to the human disease, especially with respect to preventative versus therapeutic treatment regimens, is critically important. Most importantly, realize that hope is no substitute for hard data.

Finally, it is clear that some of the phase 3 clinical failures that we have reviewed were very unlikely to succeed. The latest trials of bapineuzumab and solanezumab provide tantalizing clues with respect to biomarker changes and clinical efficacy that remain challenging to interpret.^116^ The field also awaits with great interest the progress of the phase 3 trials of MK-8931, a Beta-amyloid cleaving enzyme inhibitor that will inhibit the production of Aβ. MRK-8931 is being tested in prodromal AD (http://ClinicalTrials.gov identifier NCT01953601), where the primary outcome measure is the change from baseline CDR-SB at 2 years, and in mild–moderate AD (ClinicalTrials.gov identifier NCT01739348), where the coprimary outcome measures are a change from baseline in ADAS-cog and ADCS-ADL at 78 weeks.

Several studies are underway that seek to affect amyloid deposition at much earlier stages of AD.^131^ The Alzheimer's Prevention Initiative has enrolled members of a large Columbian cohort who carry the E280A PS-1 mutation and will develop AD. Two hundred mutation carriers within 10 years of predicted cognitive decline will receive either crenezumab, an anti-Aβ antibody, or placebo for 5 years, with the primary endpoint being a composite cognitive test. The Dominantly Inherited Alzheimer's Network includes 160 FAD mutation carriers who are cognitively normal, or with very mild memory complaints who will receive gantenerumab, an anti-Aβ antibody, solanezumab, or placebo for 2 years followed by a biomarker study to select the most efficacious drug for a further 3-year trial with a cognitive endpoint. The Anti-Amyloid Treatment in Asymptomatic Alzheimer's Disease study will recruit 1,000 cognitively normal individuals who have tested positive on amyloid PET brain scans. They will receive solanezumab for 3 years, followed by a 2-year extension period with the Preclinical Alzheimer's Cognitive Composite test as a primary outcome measure. These studies will provide a wealth of cognitive and biomarker data.

These trials, and the hints of efficacy in mild AD demonstrated by solanezumab, provide sustenance to those drug developers and scientists who believe that the amyloid hypothesis has yet to be tested and that ultimately the field will refute the null hypothesis to provide effective therapies for this devastating disease.
